# A theory driven, pragmatic trial implementing changes to routine antenatal care that supports recommended pregnancy weight gain

**DOI:** 10.1186/s12884-022-04750-8

**Published:** 2022-05-18

**Authors:** Susan de Jersey, Taylor Guthrie, Leonie Callaway, Jeanette Tyler, Karen New, Jan Nicholson

**Affiliations:** 1 Department of Nutrition and Dietetics, Royal Brisbane and Women’s Hospital, Metro North Hospital and Health Service, Butterfield Street Herston, Brisbane, QLD 4029 Australia; 2 Centre for Clinical Research and Perinatal Research Centre, Faculty of Medicine, The University of Queensland, Brisbane, QLD 4029 Australia; 3 Women’s and Newborn Services, Royal Brisbane and Women’s Hospital, Metro North Hospital and Health Service, Brisbane, QLD 4029 Australia; 4 School of Clinical Medicine, Faculty of Medicine, The University of Queensland, Brisbane, QLD 4029 Australia; 5 Academic Consultant, Healthcare Evidence and Research, Brisbane, QLD 4006 Australia; 6 Judith Lumley Centre, La Trobe University, Bundoora, VIC 3086 Australia; 7 School of Early Childhood and Inclusive Education, Faculty of Education, Queensland University of Technology, Kelvin Grove, QLD 4059 Australia

**Keywords:** Pragmatic trial, Weight, Pregnancy, Implementation, Pregnancy weight gain chart

## Abstract

**Background:**

Prevention of weight gain outside recommendations is a challenge for health services, with several barriers to best practice care identified. The aim of this pragmatic implementation study with a historical control was to examine the impact of implementing a service wide education program, and antenatal care pregnancy weight gain chart combined with brief advice on women’s knowledge of recommended gestational weight gain (GWG), the advice received and actual GWG.

**Methods:**

The PRECEDE PROCEED Model of Health Program planning guided intervention and evaluation targets and an implementation science approach facilitated service changes. Pregnant women < 22 weeks’ gestation attending the antenatal clinic at a metropolitan birthing hospital in Australia were recruited pre (2010, *n* = 715) and post (2016, *n* = 478) implementation of service changes. Weight measurements and questionnaires were completed at recruitment and 36 weeks’ gestation. Questionnaires assessed advice received from health professionals related to healthy eating, physical activity, GWG, and at recruitment only, pre-pregnancy weight and knowledge of GWG recommendations.

**Results:**

Women who correctly reported their recommended GWG increased from 34% (pre) to 53% (post) (*p* < 0.001). Between pre and post implementation, the advice women received from midwives on recommended GWG was significantly improved at both recruitment- and 36-weeks’ gestation. For normal weight women there was a reduction in GWG (14.2 ± 5.3 vs 13.3 ± 4.7 kg, *p* = 0.04) and clinically important reduction in excess GWG between pre and post implementation (31% vs 24%, *p* = 0.035) which remained significant after adjustment (AOR 0.53 [95%CI 0.29–0.96]) (*p* = 0.005).

**Conclusions:**

Service wide changes to routine antenatal care that address identified barriers to supporting recommended GWG are likely to improve the care and advice women receive and prevent excess GWG for normal weight women.

**Supplementary Information:**

The online version contains supplementary material available at 10.1186/s12884-022-04750-8.

## Introduction

Approximately 70% of women gain weight outside of current recommendations during pregnancy [1]. Inadequate gestational weight gain (GWG) occurs in approximately a quarter of pregnancies and is associated with an increased risk of small for gestational age babies and preterm birth [1]. Excess weight gain occurs in almost half [1] of pregnancies and is associated with large for gestational age births, macrosomia [1] and future obesity in mothers [2] and their offspring [3].

While women report wanting to be provided with advice about GWG [4], this advice is seldom received or accurate [5, 6]. Furthermore, women appear to have poor knowledge about weight gain recommendations [6, 7], which can impact on actual weight gain in pregnancy [5, 7].

In a trial setting routine weight monitoring as a stand-alone measure appears to be of little value [8]. However, diet and physical interventions coupled with routine weight monitoring appear to have a small impact on reducing excess GWG [9]. In an effort to support women’s long term wellbeing, advice and support around recommended weight gain is considered part of good clinical practice. Clinical practice guideline recommendations suggest women should be offered the opportunity to be weighed and provided with appropriate advice to support recommended weight gain throughout pregnancy [2, 10]. However, it is unclear whether clinical guideline recommendations impact on the provision of routine care delivery, women’s knowledge of weight gain recommendations and the amount of weight women gain during pregnancy.

There is a pressing need to implement and evaluate routine health care service strategies that address identified barriers to the provision of best practice that supports recommended pregnancy weight gain and management [11]. Recently, the integration of pregnancy weight gain charts into routine clinical care demonstrated that while implementation of the resources needed to guide conversations and track weight was suboptimal, it was feasible to implement changes to service delivery that were well received by women [12]. The objective of this pragmatic study was to examine the impact of the implementation of a service wide education program, and antenatal care pregnancy weight gain chart combined with brief advice on women’s knowledge of recommended GWG, the advice received and their actual GWG.

## Methods

### Study design

This was a pragmatic study with a prospective pre-post health services research study design. This study was approved by the Royal Brisbane and Women’s Hospital (HREC/14/QRBW/491) and Queensland University of Technology Human Research Ethics Committee (1,500,000,362).

The PRECEDE PROCEED model of Health Program Planning [13] was used to guide pre-implementation assessment, intervention development, implementation and evaluation of changes to routine antenatal care. The PRECEDE component of the PRECEDE PROCEED Model of Health Program Planning [13] was used as a framework to undertake assessment of the factors influencing GWG. The first three phases involved the New Beginnings Healthy Mothers and Babies Study (“New Beginnings”), a prospective observational study examining influences on weight gain and lifestyle behaviours in pregnancy and post-partum, and provided the pre-implementation data to guide the planning and development of further intervention. The New Beginnings study identified the need to provide women with antenatal care that supported recommended pregnancy weight gain [4, 6, 14–16]. Both health professionals and pregnant women were identified as populations to target, ensuring health cognitions of individuals that influenced behaviour change were accommodated [16].

The PROCEED Component of the model was then applied to align potential intervention strategies to accommodate organisational resources and priorities (Phase 4), implement and evaluate these strategies (Phases 5–7) [13]. Data from the New Beginnings Study was presented at a workshop of multidisciplinary service leaders and executives to prioritise intervention strategies. At the time there were no resources for new services. Therefore, low cost interventions were prioritised that included the introduction of routine weight monitoring into antenatal care and health professional education on supporting recommended pregnancy weight gain.

### Context and service change implementation

The setting for this study was a metropolitan birthing facility with approximately 4500 births per year. The range of antenatal care models offered included midwifery group practice, birth centre care, team midwifery care, shared care with a General Practitioner (GP), a high-risk obstetric care, and a dedicated Aboriginal and Torres Strait Islander continuity of care model. All women had a first hospital visit with a midwife at 14–18 weeks’ gestation and subsequent antenatal care depended on the model of care. Hospital based models followed the minimum antenatal care schedule outlined within the National Pregnancy Care Guidelines [17] which involved monthly appointments until 28 weeks gestation, fortnightly until 36 weeks and then weekly until delivery. The GP shared care model involved hospital antenatal care visits at 14–18 weeks, 30 weeks and 36 weeks with a midwife and one obstetric visit at 20–24 weeks for pregnancies up until 40 weeks. Overdue pregnancies (40 + to 42 weeks) had scheduled obstetric weekly visits until delivery.

A facilitated implementation approach was used where a multidisciplinary working group was established with a midwifery and dietetic lead to guide changes to routine care. Strategies used to support implementation were mapped to the Expert Recommendations for Implementing Change (ERIC) compilation [18]. Sixteen discrete implementation strategies were used to support practice change including “assessing readiness and identify barriers and facilitators”, “facilitation” and “remind clinicians” (See Additional File 1). Being guided by implementation science principles a barrier assessment to the implementation of routine weight monitoring and a health professional education was undertaken through focus groups with midwives [19]. Multifaceted changes within antenatal care were made, the elements of which have been previously described in detail [12, 20, 21]. In summary, there was a need identified for a resource to support the implementation of routine weight monitoring. Pregnancy weight gain charts were developed [12]. All midwives attended mandatory training [21]. Obstetric and medical staff were provided with in-service education at existing education meetings, however this was not considered mandatory to attend. Community GP’s were provided with education through a hospital education day. Educational videos were available on computers in all work areas, and weighing scales were provided in all outpatient clinic rooms.

Pregnancy weight gain charts were to be commenced for all pregnant women at first hospital visit with a brief intervention advice framework [22] applied during routine antenatal midwifery consultations.

### Participants and data collection

The participants and recruitment processes for the pre-implementation New Beginnings study participants (2010) have been previously described [4, 6]. In brief, a consecutive sample of eligible women were recruited via mail or in person in a metropolitan antenatal care facility in Australia at <20 weeks gestation between August 2010 and January 2011 [6]. All women referred for antenatal care were eligible except those who had insufficient English language skills to complete questionnaires and those with pre-existing Type 1 or 2 diabetes [6]. Women who delivered prior to 32 weeks gestation or had an infant with major health concerns were withdrawn from the study [6]. Data already collected were retained.

The post implementation cohort (2016) were part of the Healthy Pregnancy Healthy Baby study. Recruitment occurred between November 2015 and January 2016 and has been previously described [12]. In brief a consecutive unselected sample of pregnant women who were less than 20 weeks gestation were recruited in the waiting room of the antenatal clinic at the same hospital the New Beginnings Study was conducted [12]. A research staff member approached the woman at their “first visit” appointment and asked if they would like to participate in a study evaluating the provision of health advice and weight monitoring during pregnancy [6].

All women in both cohorts provided written informed consent to participate. At their first visit women completed a questionnaire assessing pre-pregnancy weight and demographic characteristics and had their height measured. At their 36 week visit they were weighed and completed the second questionnaire. Both questionnaires assessed the advice women received in relation to healthy eating, physical activity and weight gain. Independent research staff not involved in clinical care delivery recruited women and collected data.

### Measures

#### GWG knowledge

Participants knowledge at first hospital visit was measured by categorising their reported recommended gestational weight gain value as correct or incorrect (including unsure) based on the relevant IOM guidelines recommendations for their pre-pregnancy BMI.

#### Advice received

Four items each assessed the frequency of receiving health professional advice for adequate weight gain, healthy eating and physical activity in pregnancy. Items were based on those developed for the assessment of social support [23, 24] and modified to reflect health professional specific support and advice in relation to recommended pregnancy weight gain [4]. The instrument was reviewed by an expert panel including an expert in questionnaire development and research methods, an expert in health promotion theory, a maternal health dietitian and an obstetric physician to determine content and face validity. Pilot testing was conducted under the same conditions for study administration to identify items that lacked clarity, ensure instructions, content and layout were acceptable and assess practical issues with administration [6]. Items were rated on a five-point Likert scale and asked at first visit and 36 weeks’ gestation. In the pre implementation cohort these items were asked in relation to health professional advice, whereas in the post-implementation cohort the items were asked in relation to advice received separately from doctors and midwives as a large focus of the implementation was on midwifery staff practices. Responses were highly skewed and were dichotomised for the analyses (never/rarely, vs. sometimes/usually/always) to reflect the desired frequency of health professional advice.

#### Gestational weight gain

Self-reported pre-pregnancy weight and measured height were used to calculate pre-pregnancy BMI. World Health Organization (WHO) classifications were used to categorise BMI in kg/m^2^: underweight < 18.5; normal weight 18.5–24.9; and overweight ≥ 25.0 (comprising pre-obese 25.0- 29.9 and obese ≥ 30.0) [25].

Total GWG was the difference between measured weight at the 36 week visit and pre-pregnancy weight self-reported at first visit weeks. Excess GWG gain was defined by the upper limit of IOM guidelines for each pre-pregnancy BMI category for single (underweight > 18 kg, normal weight > 16 kg, pre-obese > 11.5 kg, obese > 9 kg) [2], and multiple-fetus pregnancies (> 25 kg for normal weight and underweight, > 23 kg for pre-obese and > 19 kg for obese women) [2].

### Statistical analysis

Analyses were performed using the Statistical Package for Social Sciences (Version 26: SPSS Inc., Chicago, IL, USA). Continuous variables were examined for normality, using descriptive statistics and histograms. Normality was established if the following criteria were met: mean within 10% of median; minimum and maximum approximately mean ± 3 standard deviations; skewness and kurtosis both within ± 3, and a roughly symmetrical histogram [4]. Mean and standard deviation (mean (SD) are reported for normally distributed data; median and interquartile range (median [IQR]) for skewed data. Descriptive statistics were used to examine population characteristics and outcomes. Difference between groups used t-tests or chi squared for continuous and categorical variables respectively. Appropriate non-parametric tests were used for non-normally distributed data. Logistic regression, stratified for WHO pre-pregnancy BMI classification [25] were used to compare excess GWG between the pre and post implementation cohorts controlling for gestation at final weight measurement, age, education, number of antenatal visits and language spoken at home.

In the pre-implementation cohort, there were 13 women with a multiple pregnancy at 16 weeks and four of these provided a weight measure at 36 weeks’ gestation. In the post implementation cohort, there were seven women with a multiple pregnancy at 16 weeks and four of these provided a weight measurement at 36 weeks’ gestation. Analysis of GWG was conducted with and without multiple pregnancies and did not alter interpretation of results; they were therefore included in the presented data.

**Fig. 1:**
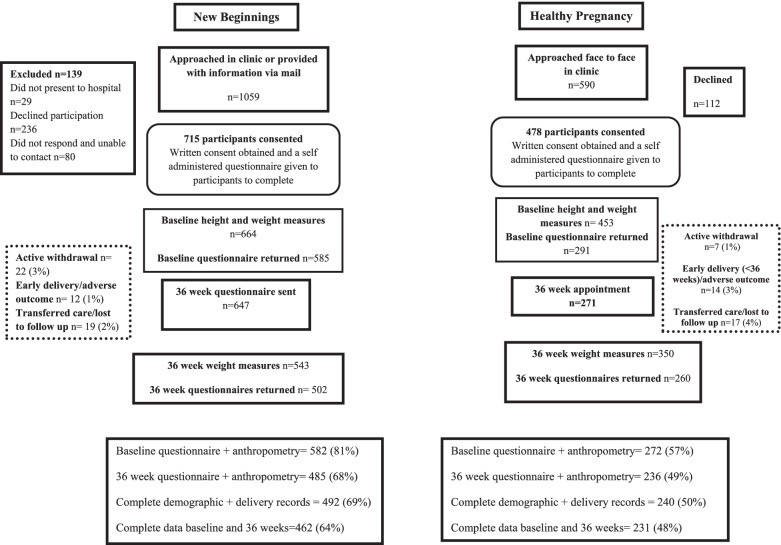
Progression of participants through the New Beginnings and Healthy Pregnancy study time points from recruitment through to 36 week follow up

Gestational weight gain could not be calculated for women who delivered before 36 weeks. These women were excluded from analyses involving GWG, and advice received at 36 weeks but were included in analysis relating to changes in GWG knowledge. The criterion for statistical significance was set at *p* < 0.05 (two tailed) for all analyses. All available data were used in analysis, no data were imputed. There was variable missing data for each time point, for each cohort (as outlined in Fig. 1). Women who gave birth prior to 36 weeks’ gestation were excluded from GWG analysis, however all data provided in relation to other measures were retained and analysed. Those who were retained in the study at 36 weeks, provided a questionnaire and weight measurement were not significantly different from those who did not complete for pre-pregnancy BMI, education status, language spoken at home, or age.

## Results

Both the pre [16] and post [12] implementation participants have been previously described. Briefly, in the pre-implementation a total of 715 from 1,059 eligible women (67%) consented to participate, and post implementation a total of 478 women consented to participate from 590 approached (81%). Figure 1 demonstrates the flow of participants from each cohort across the two study time points.

Participant characteristics of both study cohorts are described in Table 1 with post implementation participants on average 1 year older.

**Table 1:** Participants characteristics from the New Beginnings (2010) and Healthy Pregnancy (2016) Study Cohorts

Characteristic		Pre- implementation cohort (2010) *n* = 492	Post- implementation cohort (2016) *n* = 240	*p* value
Age (years)^a^	Mean ± s.d. (range)	30 ± 5.2	31 ± 5.4	** < 0.001**
Parity n (%)^b^	Nulliparous	353 (60.5)	158 (55.4)	0.151
Education n(%)^b^	Year 12 or less	129 (22.2)	34 (11.9)	**0.001**
	Trade/certificate/diploma	192 (33.0)	98 (34.3)	
	University degree	261 (44.8)	154 (53.8)	
Household Income $Au	< 50 000 pa	111 (19.1)	38 (13.4)	**0.005**
	50- 100000pa	249 (42.9)	108 (38)	
	100 000 + pa	143 (24.6)	101 (35.6)	
Employment n (%)^b^	Full time	274 (47.0)	149 (52.3)	0.304
	Part time/casual	167 (28.6)	77 (27.0)	
	Not working	142 (24.4)	59 (20.7)	
Language at home (%)^b^	English	502 (86.3)	233 (80.3)	**0.024**
Marital status n (%)^b^	Married/defacto	551 (94.5)	267 (93.7)	0.623
BMI (kg/m^2^)^a^	Mean ± s.d. (range)	24.3 ± 5.1	24.7 ± 4.6	0.236
Weight status n (%)(BMI kg/m^2^)	< 18.5	39 (5.9)	25 (5.5)	0.525
	18.5–24.9	403 (60.7)	257 (56.7)	
	25–29.9	141 (21.2)	107 (23.6)	
	> 30.0	81 (122)	64 (14.1)	
Overweight category n (%)	> 25 kg/m^2^	222 (33.4)	170 (37.7)	0.144
Gestational age at first hospital visit	Weeks (s.d.)	16.7 (2.2)	16.9 (1.9)	0.093
Gestational age at delivery	Weeks (s.d.)	39.6 (1.6)	39.2 (1.9)	< **0.01**
Number of antenatal visits	2–4	14 (2.2)	4 (0.8)	**0.004**
	5–7	67 (10.4)	27 (5.7)	
	8 or more	564 (87.4.4)	443 (93.5)	

^a^t-test for comparison

^b^Pearson’s chi squared test for comparison

Compared to pre data, there was a significant increase in the proportion of women who could correctly report their GWG recommendations 34% (pre) vs 53% (post) (*p* < 0.001) which remained significant after controlling for education in the post implementation group. The greatest knowledge increase was observed for women without a university education where those correctly identifying GWG recommendations increased from 28% (pre) to 55% (post) (compared to university educated women, 41%, pre to 51% post). The misestimates of the recommended weight gain improved at both the lower and upper end of the range in the post cohort, supporting this improvement in knowledge as outlined in Table 2.

**Table 2:** Institute of Medicine (IOM) [2] recommended weight gain ranges for body mass index (BMI) categories^f^ and participant reported ranges

		Pre-implementation cohort (*n* = 569)		Post-implementation cohort (*n* = 270)	
Pre-pregnancy BMI category	IOM recommended GWG	GWG range matched recommended n (%)	Lowest and highest reported GWG	GWG range matched recommended n (%)	Lowest and highest reported GWG
Underweight^a^	12.5–18.0	11 (33)	7–18	3 (18)	11–20
Normal weight^b^	11.5–16.0	145 (42)	0–22	90 (55)	6–20
Pre-obese^c^	7.0–11.5	21 (17)	2–25	33 (57)	6–18
Obese^d^	5.0–9.0	15 (23)	0–16	17 (46)	5–15
All women	N/A	34%	N/A	53%	N/A

*BMI* Body mass index

^**a**^underweight = BMI < 18.5 kg/m^2^

^**b**^normal weight = BMI 18.5–24.9 kg/m^2^

^**c**^pre-obese = BMI 25.0–29.9 kg/m^2^

^**d**^obese = BMI ≥ 30.0 kg/m^2^

^f^based on measured height at 16 weeks and self-reported pre-pregnancy weight

Table 3 outlines the levels of advice women reported receiving from their health professional after the implementation in relation to recommended pregnancy weight gain, healthy eating, and physical activity. Between pre and post implementation the advice women reported receiving from midwives in relation to recommended pregnancy weight gain was significantly improved at both 16- and 36-weeks’ gestation. Significant improvements were reported for recommended weight gain advice from doctors at 36 weeks only, and this was not as marked as for the midwives. Changes in specific advice about physical activity and healthy eating were inconsistent across time points and health professional group (Table 3). Improvements mostly occurred at 16 weeks (rather than 36 weeks) and these were more often by midwives than doctors.

**Table 3:** Women reporting sometimes/usually/always being provided with advice from health care professionals, (2010) and, Doctors and Midwives (2016) relating to supporting recommended pregnancy weight gain at 16 and 36 weeks gestation [percentage (count)]

Domain	Supportive advice item	16 weeks HCP sometimes/usually/always Pre (*n* = 575) % (n)	16 weeks Midwives sometimes/usually/always Post (*n* = 274) % (n)	16 weeks Doctor sometimes/usually/always Post (*n* = 274) % (n)	36 weeks HCP sometimes/usually/always Pre (*n* = 492) % (n)	36 weeks Midwives sometimes/usually/always Post (*n* = 240) % (n)	36 weeks Doctors sometimes/usually/always Post (*n* = 240) % (n)
The health care professionals who have cared for me since I became pregnant …							
Recommended pregnancy weight gain	encourage me to weigh myself regularly	25 (141)	54 (147)**	27 (73)	11 (53)	51 (130)**	28 (68)**
	check how much weight I have gained	59 (338)	84 (230)**	57 (158)	35 (174)	88 (223)**	66 (158)**
	offer advice about how much weight I should gain in my pregnancy	39 (222)	74 (202)**	43 (119)	26 (128)	69 (174)**	47 (112)**
	offer me advice about how to gain the right amount of weight in my pregnancy	29 (164)	53 (144)**	30 (82)	17 (81)	49 (125)**	31 (74)**
Healthy eating	ask me about the foods I eat	43 (247)	46 (128)	48 (135)	39 (191)	47 (119)*	43 (106)
	encourage me to eat healthy foods	64 (373)	65 (181)	66 (186)	58 (287)	63 (160)	61 (148)
	give advice about the amount of food to eat	29 (169)	36 (102)*	32 (91)	21 (104)	33 (83)**	27 (65)
	give advice about how to plan and prepare healthy food	16 (95)	30 (84)**	22 (61)	13 (65)	17 (42)	14 (33)
Physical activity	ask me about the physical activity I do	39 (226)	39 (108)	48 (136)*	42 (206)	49 (124)	45 (109)
	encourage me to be physically active	47 (273)	51 (141)	57 (159)*	50 (243)	59 (151)*	54 (131)
	offer advice about how to include physical activity in my day	23 (135)	31 (85)*	29 (80)	21 (101)	31 (80) *	22 (53)

*HCP* Health care professional

^**^significant difference between pre and post implementation data, *p* < 0.001 at same time point

^*^significant difference between pre and post implementation data *p* < 0.05 at same time point

The prevalence of excess GWG was 38% pre and 36% post implementation. After controlling for baseline differences between pre and post implementation cohorts and the gestation at final weight measurement as a group the cohorts did not differ on the proportion of excess GWG (*p* = 0.06). Table 4 outlines total GWG for each WHO pre-pregnancy BMI category. For women with a pre-pregnancy BMI in the under-weight, pre-obese and obese category there were no differences in total or excess GWG between pre and post implementation. For normal weight women unadjusted and adjusted comparisons for total and excess GWG were significantly different. There was a 1 kg reduction in GWG (14.2 ± 5.3 vs 13.3 ± 4.7 kg, *p* = 0.04) and a clinically important reduction in the proportion with excess GWG between pre and post implementation (31% vs 24%, *p* = 0.035, AOR 0.53 [95%CI 0.29–0.96]) which remained significant after adjustment (*p* = 0.005).

**Table 4:** Total gestational weight gain (GWG) according to the pre-pregnancy weight status^**c**^ (WHO Classification) of 2010 and 2016 participants [Mean ± s.d. (range)^d^

**Characteristic**	**Underweight**^**+**a^ **2010 6% (*n* = 39)**	**Underweight**^**+**a^ **2016 5% (*n* = 25)**	**Normal weight**^**+**a^ **2010 61%(*n* = 403)**	**Normal weight**^**+**a^ **2016 57% (*n* = 257)**	**Pre-obese**^**+**a^ **2010 21% (*n* = 141)**	**Pre-obese**^**+**a^ **2016 24% (*n* = 107)**	**Obese**^**+**a^ **2010 12% (*n* = 81)**	**Obese**^**+**a^ **2016 14% (*n* = 64)**
Total GWG at about 36^b^ weeks gestation (kg)	14.3 ± 4.3	13.7 ± 5.0	14.2 ± 5.3*	13.3 ± 4.7*	13.8 ± 6.8	13.1 ± 5.3	7.5 ± 8.7	8.6 ± 8.5
	(7.5–23.0)	(6–26)	(-4.0–38.4)	(-7.6–26.9)	(-3.0–35.6)	(2.3–30.1)	(-10.6–24.0)	(-10.1–22.4)
	[*n* = 27]	[*n* = 20]	[*n* = 338]	[*n* = 202]	[*n* = 116]	[*n* = 84]	[*n* = 62]	[*n* = 44]

^a^measured height and self reported pre-pregnancy weight

^b^measured weight

^c^World Health Organization weight status categories

^d^comparisons adjusted for weeks gestation at final weight measure (weeks), age (years), education (university degree vs. less than university degree), number of antenatal visits, and language spoken at home, (English vs other); ^+^underweight, pre-pregnancy body mass index (BMI) < 18.5 kg/m^2^; normal weight pre-pregnancy BMI 18.5–24.9 kg/m^2^; pre-obese pre-pregnancy BMI 25.0–29.9 kg/m^2^; obese pre-pregnancy ≥ 30 kg/m^2^

^*^2010 vs 2016 data significantly different *p* < 0.05

## Discussion

This theory informed; pragmatic study evaluated service wide changes to support the delivery of best practice care in relation to recommended GWG. It demonstrated that women reported improved advice about recommended weight gain from midwives, improved knowledge of GWG recommendations, and reduced excess GWG in women who started pregnancy a normal weight. However, advice from doctors and midwives relating to healthy eating and physical activity did not consistently change. For underweight, pre-obese and obese women this low intensity, “one size fits all” approach was insufficient to impact on GWG and perhaps doesn’t recognise the underlying complexities that may be associated with weight.

Advice women reported receiving relating to GWG, both generally and specifically improved as a result of the practice changes, particularly in relation to midwives. The improvements in advice observed are likely to reflect real practice change as they are based on the advice women reported receiving rather than health professionals reporting their own behaviour which is common in this type of research. However, for healthy eating and physical activity, specific advice relating to how much to eat, how to prepare healthy meals, or how to include regular physical activity as part of their day was poor, with inconsistent changes. These findings mirror those of other research where women perceive advice as being too general [26] and specific details about food requirements are rarely provided [27]. In the current study it was notable that the changes were more pronounced for the midwives than for doctors. Engagement in service changes and education from midwifery staff was much higher than for doctors. The education for midwifery staff was mandatory, whereas for doctors it was more opportunistic. It is likely some of the observed differences in advice was as a result of the greater saturation of training received within the midwifery discipline than for doctors. Women often see multiple health care professionals including obstetricians, general practitioners and midwives during pregnancy, however midwives may have more capacity to counsel women on key lifestyle aspects than doctors with their approach more focussed on holistic care [27]. Further exploration is needed as to whether more specific healthy eating, physical activity and weight gain advice delivered by doctors and midwives at key visits is appropriate to their role or if greater access to other appropriate professionals such as dietitians is needed [27, 28].

This low intensity intervention incorporated into routine care resulted in a modest reduction in excess GWG for women who commenced pregnancy a normal weight. Excess GWG has been attributed to a cycle of increasing BMI in women of reproductive age, where weight retention post-partum, leads to a higher pre-pregnancy BMI at a subsequent pregnancy with associated consequences [29]. This brief intervention taking as little as 1–3 min [30] may reduce excess GWG. Excess GWG often puts women into an unhealthy weight range for the first time, with heightened risk for staying at an unhealthy weight and gaining more over time and with subsequent pregnancies [31, 32]. It has been suggested that almost nine out of ten women with a normal BMI prior to pregnancy with excess weight gain will become pre-obese or obese within 5 years post-partum [33]. With 60% of the sample in the current study, similar to state-wide estimates [34], having a normal BMI prior to pregnancy the implications for future population health of women are likely significant.

While it is clear this intervention was helpful for many normal weight women, more work needs to be done to unpack why those above or below a normal weight prior to pregnancy did not appear to benefit beyond increasing knowledge of recommendations. It was accepted that other supporting and peripheral intervention strategies may support the adoption of the desired practice changes. It has previously been demonstrated that while women above a normal weight have strong intentions to manage their weight during pregnancy, they experience greater barriers and have a lower confidence for overcoming challenges [15]. Furthermore, health cognitions appear to be associated with excess GWG and these differ according to pre-pregnancy BMI [16]. Previous experiences with weight management, weight potentially being a triggering factor and previous stigmatising experiences are likely to be more common in women outside of a body weight considered normal [35, 36]. Collectively this evidence suggests the need for more targeted interventions and greater time investment during consultations to understand women’s previous experiences and provide individualised support. These aspects of developing a therapeutic relationship with women may be difficult to achieve through brief interventions, particularly with a lack of continuity in antenatal care [19].

The improvements were observed in the context of sub-optimal implementation of some aspects of the intervention, in particular the pregnancy weight gain charts. [12] Further work needs to explore if more consistent and accurate use of the weight gain charts results in further improvements in the outcomes observed in this study.

### Strengths and limitations

The findings of this research should be considered in the context of several strengths and limitations. This study was not a randomised trial. As a pragmatic trial, changes within the broader community and hospital over time may have impacted study findings. However, the applied nature in routine care demonstrated the feasibility of the small changes having a positive impact. Between the two study periods there were changes to health service policy around who was accepted to birth within the facility whereby women who were not eligible for publicly provided health services (“Medicare ineligible”), and those from outside the hospital catchment were no longer accepted in the post-implementation period, impacting on language spoken at home and education differences. There were also antenatal care scheduling improvements where a greater proportion of women had 8 or more appointments post implementation. However, these differences were small and were accounted for in statistical analysis. Furthermore, the pre implementation study sample was comparable to the broader pregnant population from which they were recruited [6, 37], giving strength to the representativeness of the cohort. A consideration is pre-implementation women were asked to report the advice received from health professionals, however post implementation this was separated to midwives and doctors due to the more intensive nature of implementation strategies focussing on midwifery practice. Due to the different pre-comparison group, interpretation of changes in advice needs to be undertaken with caution. However, the magnitude of changes and improvements across knowledge for women given confidence in the positive changes in advice women report receiving. Self-reports from the women indicate changed practices specifically relating to GWG, and the greatest improvements in GWG knowledge was observed in less educated women, so this gives greater confidence that the difference in GWG from pre to post groups is at least partly if not mostly due to the intervention. The post implementation cohort had a higher consent rate, however a greater proportion of missing data at follow up. These differences were because of different staffing between recruitment and follow up which meant more intensive recruitment but more follow up appointments missed by study staff, impacting on statistical power for sub-group analysis. An important strength of this research was considering women’s pre-pregnancy BMI, to allow identification of who did not benefit from this intervention. For these women, given the combination that midwives and doctors were not good at giving specific advice on eating and exercise, it is likely that early access to appropriate allied health professionals may be required to achieve healthier GWG. Another key strength of this work was the strong theoretical foundation to intervention strategy development and the implementation science approach to integrating service changes based on the barrier identification and problem assessment. Sixteen discrete implementation strategies were used to support practice change.

While the use of multiple implementation strategies strengthens the chance of health service changes being embedded into routine care, further evaluation is required to determine if these practice improvements are sustained longer term.

## Conclusion

Using theory and implementation science to scale and spread this approach to integrating low intensity strategies into routine care that support recommended pregnancy weight gain may improve the care and advice women receive and reduce excess GWG for normal weight women.

## Supplementary Information

**Additional file 1.**

## Data Availability

The datasets generated and analysed during the current study are not publicly available do to ethical restrictions but are available from the corresponding author on reasonable request.

## References

[1] Goldstein RF, Abell SK, Ranasinha S, Misso M, Boyle JA, Black MH (2017). Association of gestational weight gain with maternal and infant outcomes: a systematic review and meta-analysis. JAMA.

[2] Institute of Medicine and National Research Council. Weight gain during pregnancy: reexamining the guidelines. Washington, DC: National Academies Press; 2009. 20669500

[3] Voerman E, Santos S, PatroGolab B, Amiano P, Ballester F, Barros H (2019). Maternal body mass index, gestational weight gain, and the risk of overweight and obesity across childhood: an individual participant data meta-analysis. PLoS Med.

[4] de Jersey SJ, Nicholson JM, Callaway LK, Daniels LA (2013). An observational study of nutrition and physical activity behaviours, knowledge, and advice in pregnancy. BMC Pregnancy Childbirth.

[5] Deputy NP, Sharma AJ, Kim SY, Olson CK (2018). Achieving appropriate gestational weight gain: the role of healthcare provider advice. J Womens Health.

[6] de Jersey SJ, Nicholson JM, Callaway LK, Daniels LA (2012). A prospective study of pregnancy weight gain in A ustralian women. Aust N Z J Obstet Gynaecol.

[7] Shulman R, Kottke M (2016). Impact of maternal knowledge of recommended weight gain in pregnancy on gestational weight gain. Am J Obstet Gynecol.

[8] Fealy SM, Taylor RM, Foureur M, Attia J, Ebert L, Bisquera A (2017). Weighing as a stand-alone intervention does not reduce excessive gestational weight gain compared to routine antenatal care: a systematic review and meta-analysis of randomised controlled trials. BMC Pregnancy Childbirth.

[9] Muktabhant B, Lawrie TA, Lumbiganon P, Laopaiboon M (2015). Diet or exercise, or both, for preventing excessive weight gain in pregnancy. Cochrane Database Syst Rev.

[10] Homer CS, Oats J, Middleton P, Ramson J, Diplock S (2018). Updated clinical practice guidelines on pregnancy care. Med J Aust.

[11] Harrison CL, Skouteris H, Boyle J, Teede HJ (2017). Preventing obesity across the preconception, pregnancy and postpartum cycle: implementing research into practice. Midwifery.

[12] de Jersey S, Guthrie T, Tyler J, Ling WY, Powlesland H, Byrne C (2019). A mixed method study evaluating the integration of pregnancy weight gain charts into antenatal care. Matern Child Nutr.

[13] Green LWKM (2005). Health program planning: an educational and ecological approach.

[14] de Jersey SJ, Callaway LK, Daniels L, Nicholson J (2015). Weight-related risk perception among healthy and overweight pregnant women: a cross-sectional study. J Perinatol.

[15] de Jersey SJ, Mallan K, Callaway L, Daniels LA, Nicholson JM (2017). A cross sectional comparison of predisposing, reinforcing and enabling factors for lifestyle health behaviours and weight gain in healthy and overweight pregnant women. Matern Child Health J.

[16] de Jersey SJ, Mallan KM, Callaway LK, Daniels LA, Nicholson JM (2017). Prospective relationships between health cognitions and excess gestational weight gain in a cohort of healthy and overweight pregnant women. J Acad Nutr Diet.

[17] Department of Health. Clinical Practice Guidelines: Pregnancy Care. Canberra: Australian Government Department of Health; 2018.

[18] Powell BJ, Waltz TJ, Chinman MJ, Damschroder LJ, Smith JL, Matthieu MM (2015). A refined compilation of implementation strategies: results from the expert recommendations for Implementing Change (ERIC) project. Implement Sci.

[19] Guthrie TM, de Jersey SJ, New K, Gallegos D (2020). Midwife readiness to provide woman-centred weight gain support: exploring perspectives across models of care. Women and Birth.

[20] Yim SY, Guthrie T, de Jersey SJ. Impact of service‐wide initiatives to support healthy pregnancy weight gain on weight‐related documentation. Aust N Z J Obstet Gynaecol. 2020;60(3):355–60. 10.1111/ajo.13053 31578721

[21] de Jersey SJ, Tyler J, Guthrie T, New K (2018). Supporting healthy weight gain and management in pregnancy: Does a mandatory training education session improve knowledge and confidence of midwives?. Midwifery.

[22] Glasgow RE, Davis CL, Funnell MM, Beck A (2003). Implementing practical interventions to support chronic illness self-management. Jt Comm J Qual Saf.

[23] Chang M-W, Brown R, Nitzke S (2008). Scale development: factors affecting diet, exercise, and stress management (FADESM). BMC Public Health.

[24] Sallis JF, Grossman RM, Pinski RB, Patterson TL, Nader PR (1987). The development of scales to measure social support for diet and exercise behaviors. Prev Med.

[25] World Health Organization. Obesity: preventing and managing the global epidemic. Geneva: World Health Organization; 2000. 11234459

[26] Duthie EA, Drew EM, Flynn KE (2013). Patient-provider communication about gestational weight gain among nulliparous women: a qualitative study of the views of obstetricians and first-time pregnant women. BMC Pregnancy Childbirth.

[27] Morris J, Nikolopoulos H, Berry T, Jain V, Vallis M, Piccinini-Vallis H (2017). Healthcare providers’ gestational weight gain counselling practises and the influence of knowledge and attitudes: a cross-sectional mixed methods study. BMJ Open.

[28] Wilkinson SA, Donaldson E, Willcox J (2020). Nutrition and maternal health: a mapping of Australian dietetic services. BMC Health Serv Res.

[29] Gilmore LA, Klempel-Donchenko M, Redman LM. Pregnancy as a window to future health: Excessive gestational weight gain and obesity. Seminars in Perinatology. 2015;39(4):296–303. 10.1053/j.semperi.2015.05.009 PMC4516569 26096078

[30] Daley AJ, Jolly K, Jebb S, Roalfe A, Mackillop L, Lewis A (2015). Effectiveness of regular weighing, weight target setting and feedback by community midwives within routine antenatal care in preventing excessive gestational weight gain: randomised controlled trial. BMC obesity.

[31] Gunderson EP, Abrams B (1999). Epidemiology of gestational weight gain and body weight changes after pregnancy. Epidemiol Rev.

[32] Linné Y, Dye L, Barkeling B, Rössner S (2004). Long-term weight development in women: a 15-year follow-up of the effects of pregnancy. Obes Res.

[33] Davis EM, Stange KC, Horwitz RI (2012). Childbearing, stress and obesity disparities in women: a public health perspective. Matern Child Health J.

[34] Queensland Health. Queensaldn Perinatal Statistics Annual Report 2019. Brisbane: Queensland Health; 2021.

[35] Vanstone M, Sadik M, Van Blyderveen S, Biringer A, Sword W, Schmidt L (2020). Competing priorities: a qualitative study of how women make and enact decisions about weight gain in pregnancy. BMC Pregnancy Childbirth.

[36] Knight-Agarwal CR, Williams LT, Davis D, Davey R, Shepherd R, Downing A, et al. The perspectives of obese women receiving antenatal care: a qualitative study of women’s experiences. Women and Birth. 2016;29(2):189–95. 10.1016/j.wombi.2015.10.008 26563638

[37] Queensland Health. Queensland Perinatal Statistics September 2010-February 2011. 2012.

