# Supplementary material for: A theory driven, pragmatic trial implementing changes to routine antenatal care that supports recommended pregnancy weight gain
Source: BMC Pregnancy Childbirth. 2022 May 18;22:416. doi: 10.1186/s12884-022-04750-8 (PMC9118702; doi:10.1186/s12884-022-04750-8)
Supplement: Supplementary file 1 — Additional file 1. [file 12884_2022_4750_MOESM1_ESM.docx]

Additional File 1 Expert Recommendations for Implementing Change (ERIC) (18) strategies used to facilitate changes to routine care to support recommended pregnancy weight gain.

| Strategy | Definition | Description of activities |
| --- | --- | --- |
| Assess for readiness and identify barriers and facilitators | Assess various aspects of an organization to determine its degree of readiness to implement, barriers that may impede implementation, and strengths that can be used in the implementation effort | Initial discussions with service leaders at multidisciplinary workshop  Conducted focus groups with midwifery workforce  Ongoing discussions with multidisciplinary working group throughout implementation |
| Build a coalition | Recruit and cultivate relationships with partners in the implementation effort | Multidisciplinary workshop engagement  Multidisciplinary working group  Engaged clinicians within models of care internally and GP primary health network liaison |
| Conduct educational meetings | Hold meetings targeted toward different stakeholder groups (*e.g.*, providers, administrators, other organizational stakeholders, and community, patient/consumer, and family stakeholders) to teach them about the clinical innovation | In- services to all clinicians and work areas  Mandatory training implemented for midwifery workforce |
| Conduct educational outreach visits | Have a trained person meet with providers in their practice settings to educate providers about the clinical innovation with the intent of changing the provider’s practice | Voice over PowerPoint videos added to computers in each work area  Training incorporated into GP Alignment program |
| Conduct local consensus discussions | Include local providers and other stakeholders in discussions that address whether the chosen problem is important and whether the clinical innovation to address it is appropriate | Multidisciplinary workshop discussion and options paper and gain consensus on intervention components |
| Conduct local needs assessment | Collect and analyze data related to the need for the innovation | New Beginnings Healthy Mothers and Babies Study undertaken |
| Conduct ongoing training | Plan for and conduct training in the clinical innovation in an ongoing way | Ongoing training within midwifery mandatory training, orientations of new starters and GP Alignment program |
| Develop and implement tools for quality monitoring | Develop, test, and introduce into quality-monitoring systems the right input—the appropriate language, protocols, algorithms, standards, and measures (of processes, patient/consumer outcomes, and implementation outcomes) that are often specific to the innovation being implemented | Protocol for Healthy Pregnancy Healthy Baby evaluation developed including audit protocol for pregnancy weight gain charts |
| Develop education materials | Develop and format manuals, toolkits, and other supporting materials in ways that make it easier for stakeholders to learn about the innovation and for clinicians to learn how to deliver the clinical innovation | Education materials developed including voice over Powerpoint video, verbal presentations, and great clinical questions email broadcast. |
| Distribute educational materials | Distribute educational materials (including guidelines, manuals, and toolkits) in person, by mail, and/or electronically | Education materials distributed via email, and in person at in-services/training sessions |
| Facilitation | A process of interactive problem solving and support that occurs in a context of a recognized need for improvement and a supportive interpersonal relationship | Working group co-chairs championed implementation through ongoing problem solving, engaging with staff and working group actions |
| Inform local opinion leaders | Inform providers identified by colleagues as opinion leaders or “educationally influential” about the clinical innovation in the hopes that they will influence colleagues to adopt it | Clinical leaders within obstetric, medical and midwifery workforce engaged as working group members |
| Mandate change | Have leadership declare the priority of the innovation and their determination to have it implemented | Service line executives involved in all levels of planning and intervention development. Formal memo issued from Executive Director informing of intervention implementation |
| Provide technical assistance | Develop and use a system to deliver technical assistance focused on implementation issues using local personnel | Healthy Pregnancy Health Baby working group members brought issues for discussion monthly. Group Chairs attended work area meetings to address issues and concerns |
| Provide ongoing consultation | Provide ongoing consultation with one or more experts in the strategies used to support implementing the innovation | Healthy Pregnancy Healthy Baby evaluation included consumer and staff consultation on the implementation and suggestions for change.  Clinical encouraged to email working group leads with clinical questions and feedback.  Ongoing monthly Healthy Pregnancy Healthy Baby working group meetings. |
| Remind clinicians | Develop reminder systems designed to help clinicians to recall information and/or prompt them to use the clinical innovation | Monthly emails send to clinicians during the intensive implementation phase. |
